# Design of respirable sprayed microparticles of encapsulated bacteriophages

**DOI:** 10.3389/fddev.2023.1209534

**Published:** 2023-06-14

**Authors:** Alberto Baldelli, Mingtao Liang

**Affiliations:** ^1^ Faculty of Land and Food Systems, The University of British Columbia, Vancouver, BC, Canada; ^2^ School of Biomedical Sciences and Pharmacy, University of Newcastle, Callaghan, NSW, Australia

**Keywords:** particle engineering, respiratory delivery, spraying techniques, phage therapy, bacteriophage

## Abstract

Antibiotic resistance is exponentially increasing, and the number of deaths caused by bacterial infections is expected to surge. When dealing with the respiratory system, inefficient antibiotics heighten the chance of death from bacterial infection. However, the alternatives to antibiotics are limited. Bacteriophages are a valid option since they can target a specific type of bacterium. Bacteriophages are highly specific and can avoid any side effects when delivered. However, their poor stability makes their use inefficient. Encapsulation is commonly used to protect any bioactive compound for different types of delivery. In the case of respiratory delivery, particle engineering is used to generate stable dry powders to target the nasal or lung areas. This review article provides a guideline for engineering a process of nasal dry powders of encapsulated bacteriophages.

## 1 Introduction

**FIGURE 1:**
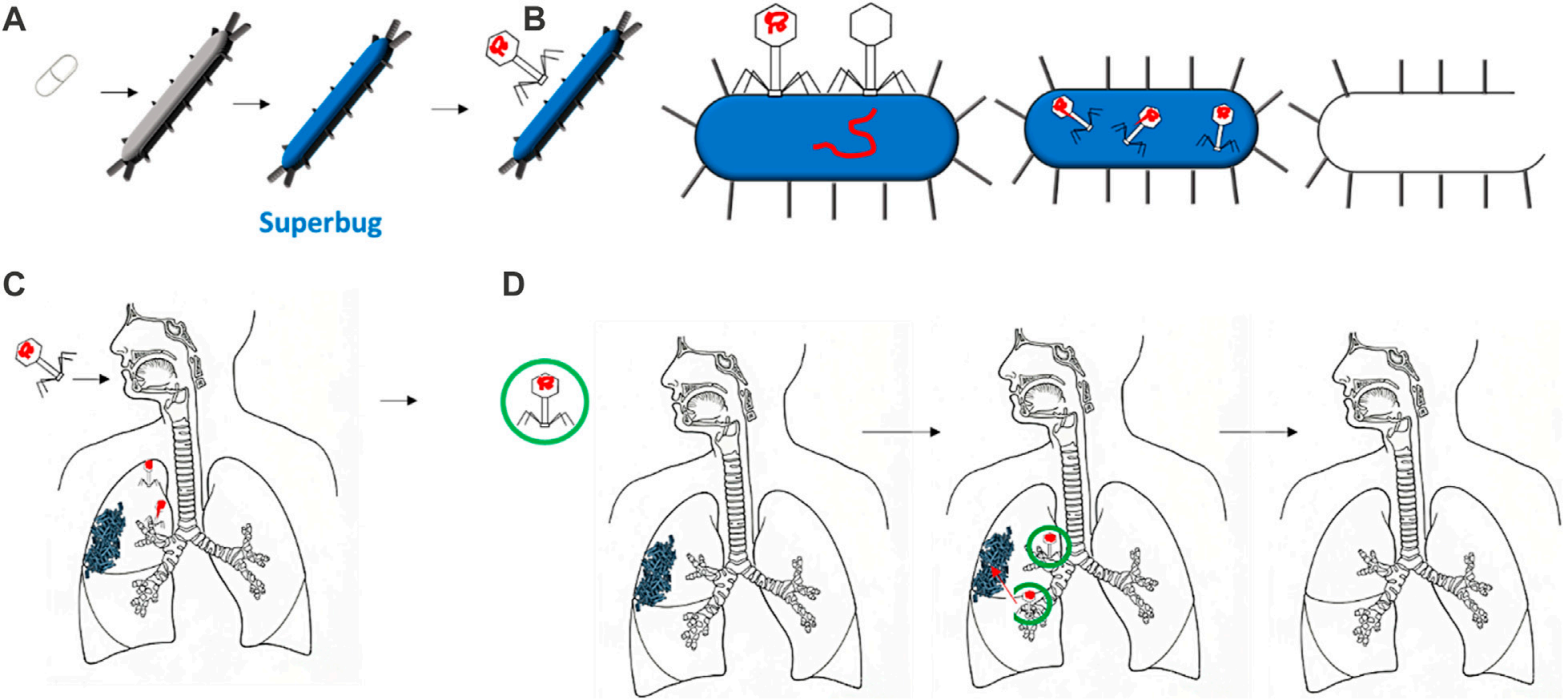
Several bacteria are growing into superbugs, mutating to resist the most common antibiotics **(A)**. Therefore, bacteriophages are increasing their effectiveness in defeating such superbugs by specifically targeting them, injecting their DNA, and reproducing in it **(B)**. The delivery of bacteriophages via the respiratory tract has shown some disadvantages, such as disrupting the bacteriophage head during delivery **(C)**. Thus, encapsulation can protect the bacteriophage once delivered, allowing it to reach the target: the bacteria or biofilms **(D)**.

Antibiotic resistance is a sword of Damocles over many lives; the research community is rightly addressing it seriously. In the United States alone, more than 2.8 million antimicrobial-resistant infections are recorded each year, from which more than 35,000 people die (Stewart and Costerton, 2001). A bacterium that learns how to defeat the attack of antibiotics is called a superbug (Figure 1A)). There are five currently known superbugs: Methicillin-resistant *Staphylococcus aureus,* extended spectrum beta-lactamase (ESBL)-producing Enterobacteriaceae*,* Vancomycin-resistant *Enterococcus,* and multidrug-resistant *Pseudomonas aeruginosa*. However, this number is expected to grow (Perez et al., 2014).

Bacteriophages—viruses that can infect and kill bacteria—are considered a promising strategy for overcoming antibiotic resistance (Eiserling, 2023). They attach to a specific type of bacterium, release their DNA, and replicate in the bacterial machinery. Later, due to several factors that differ across the type of bacterium and bacteriophage, the bacterium bursts to release virions that are ready to infect and kill other bacteria (Figure 1B) (Loc-Carrillo and Abedon, 2011). The specificity of the bacteria attacked is one of the main advantages of bacteriophages over antibiotics. Bacteriophages are considered safer since they only infect the target bacterium and have no effect on mammalian cells. Moreover, the exponential growth of bacteriophages may mean less frequent and lower doses than required for antibiotics (Sulakvelidze et al., 2001). Furthermore, the development of bacteriophages is relatively more rapid and inexpensive than antibiotic drugs (Parasion et al., 2014).

The most important concept for the success of phages in reaching bacterial targets is ensuring that a minimum inhibitory concentration is surpassed. This can be achieved by directly applying phages to the bacterium or by systemic delivery (Malik et al., 2017). Whenever delivering any bioactive compounds to the human body, large losses of compounds can occur when delivered to the respiratory tract due to degradation or aggregation. Encapsulation is the common method for enhancing compound stability and efficacy for drug delivery (Baldelli et al., 2022a). For phage delivery, the process of encapsulation involves the creation of a shell surrounding one or more bacteriophages. This shell protects the bacteriophages from external conditions and can increase adhesion to specific bacteria (Choińska-Pulit et al., 2015; Baldelli et al., 2022b). This can be extremely beneficial when bacteriophages are to reach the human lungs. The respiratory tract has evolved a mechanism to exclude anything from entering the lung. As shown in Figures 1C and D, the morphology of the airways can lead to bacteriophages being deposited in undesired locations, thus missing the target bacterium (Dasaraju and Liu, 1996). Moreover, the delivery of a free bacteriophage can trigger phage inactivation by the immune system (Żaczek et al., 2016).

Delivering bacteriophages to the respiratory system can be challenging since it can provoke different types of stress on the phages. First, a phage’s stability depends on its ionic strength; thus, any change to this parameter in the respiratory tract can generate severe damage. Second, the process of respiratory delivery can influence the osmotic pressure between the phages’ external and internal parts . Lastly, the mechanical stress involved in preparations, such as high-speed mixing, centrifugation, and spraying, can jeopardize the stability of phages by specifically deteriorating their dentures (Froman et al., 1954). Furthermore, phages, like several other treatments, can trigger the immune system of the nasal environment (Dąbrowska, 2019). Encapsulation can overcome these challenges and increase the delivery efficiency of bacteriophages to the respiratory tract.

Encapsulating a bioactive compound as dry powder can have other benefits, such as ease of use and low costs (Maa et al., 1999). Respiratory diseases such as asthma, cystic fibrosis, and chronic obstructive pulmonary diseases are currently treated using dry powders (Huang et al., 2004). Through particle engineering, scientists can engineer respirable microparticles composed of a wall material—generally a polymer of large molecular weight—and a bulk material—generally sugar or salt (Baldelli et al., 2015). The selection of the wall material influences the dimension and morphology of the microparticles, while the selection of the bulk material can impact the stability and aerosol efficiency of microparticles (Baldelli and Vehring, 2016a; Baldelli et al., 2016). Moreover, the wall material influences the location of particle deposition in the respiratory tract: a highly sticky and cohesive polymer could create aggregates that lead to an undesirable deposition of the drug delivered (Baldelli and Vehring, 2016b). In addition, the bulk material selection needs to be tailored for a specific bioactive compound. For instance, bulk materials that tend to crystallize when drying might damage the chemical structure of the bioactive compound in the inner core of the evaporating droplet (Baldelli et al., 2016; Gomez et al., 2021).

Consequently, selecting the materials and their ratios to properly encapsulate a bacteriophage is important for the efficacy and efficiency of bacteriophage delivery to the respiratory tract. In fact, if not properly planned, encapsulation can have the detrimental effect of greater deterioration of the phages. This review discusses the methods and materials for formulating respirable sprayed microparticles to encapsulate different types of bacteriophages.

## 2 Respiratory bacteria and bacteriophage therapy

### 2.1 Respiratory bacteria and antibiotic resistance

#### 2.1.1 Bacteria in the respiratory tract

**FIGURE 2:**
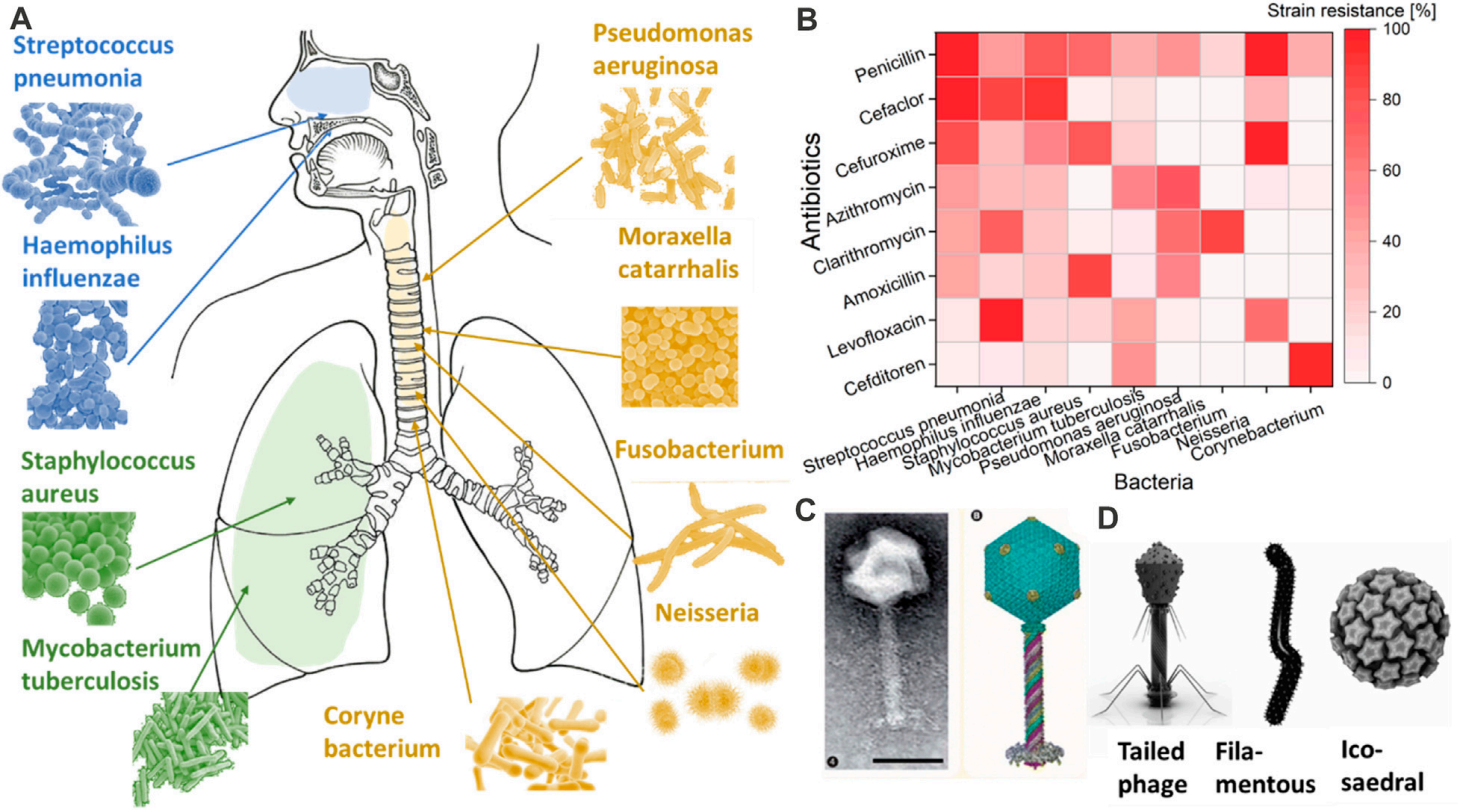
Most common types of bacteria are shown in **(A)**. The resistance of these common bacteria to the most common antibiotics is shown in **(B)** as a percentage of strain resistance. While in **(C,D)**, an image of bacteriophages of Pseudomonas and different shapes of bacteriophages are provided with the request from COPYRIGHT 2010 Future Medicine Ltd (Ceyssens and Lavigne, 2010) and Springer Link (Rohwer and Barott, 2013).

Every year, more than 13 million people are affected by respiratory infections, 11.6% of which are provoked by bacteria (Dasaraju and Liu, 1996). These numbers are expected to increase with the antibiotic resistance of bacteria. The most common bacteria that affect the respiratory tract are *Streptococcus pneumoniae, Haemophilus influenzae* and *Moraxella catarrhalis* (Bosch et al., 2013). In addition to these, *Staphylococcus aureus, Mycobacterium tuberculosis, Pseudomonas aeruginosa, Fusobacterium, Neiserria*, and *Corynebacterium* are other types of bacteria that impact different areas of the respiratory tract (Figure 2) (Prat and Lacoma, 2016). Each bacterium is acquiring resistance to the most common types of antibiotics, such as penicillin, cefaclor, cefuroxime, azithromycin, clarithromycin, amoxicillin, levofloxacin, and cefditoren (Enne et al., 2002; Akcali et al., 2005; Ceyssens and Lavigne, 2010; Hoa et al., 2010; Nie et al., 2015; Foster, 2017; Cillóniz et al., 2018; van Rijn et al., 2019; Hennart et al., 2020).

*Pneumococcus* resistance occurs especially in asymptomatic children, who can hold the bacterium for a longer time. This bacterium shows resistance against the most common antibiotics. For example, β-lactam antibiotics act against *S. aureus* by inhibiting the final steps of peptidoglycan synthesis (cell wall) by attaching to high molecular-weight penicillin-binding proteins (Feng et al., 2000). However, the bacterium can alternate its cell walls, making the binding less efficient. Macrolides antibiotics restrict protein synthesis by binding 23S ribosomal target sites in bacteria. The response of the bacterium is a ribosomal alteration by an enzyme that methylates 23S rRNA subunits and is encoded by the ermB (erythromycin resistance methylase) gene (Foster, 2017). Fluoroquinolones are probably the strongest against *Staphylococcus* by inhibiting DNA synthesis by interacting with intracellular drug targets, DNA gyrase, and topoisomerase. However, even in this case, the bacterium can modify the quinolone resistance-determining region (QRDR) (Hoe et al., 2013). Many other bacteria follow the path of *S. aureus* (Enne et al., 2002; Akcali et al., 2005; Ceyssens and Lavigne, 2010; Perez et al., 2014; Ezewudo et al., 2018; Li et al., 2018; Kabwe et al., 2019; Rubin et al., 2020). Another example is *M. tuberculosis* (Mtb), which, despite its low mutation rate, generates drug-resistant tuberculosis (TB) that has significant health and economic costs (Sudre et al., 1992). Mtb can generate pulmonary cavities unseen by immune factors and poorly accessible to antimicrobial drugs, hitherto sheltering large populations of bacteria and providing the perfect circumstances for inducing resistance (Nguyen, 2016). Therefore, highly transmissible multiresistant Mtb strains are beginning to emerge (Nguyen, 2016).

#### 2.1.2 Bacteriophages and phage therapy

Resistance to antibiotics highlights the importance of seeking alternative cures for bacterial infection, such as the use of bacteriophages.

Bacteriophages, also called ‘phages’, can be described as bacteria eaters. They have a basic structure consisting of a polyhedral head, a short collar, and a helical tail (Yap and Rossmann, 2014). Figures 2D and 2C show an example of a bacteriophage connected to *Pseudomonas*. There are three basic structural forms of bacteriophage: 1) an icosahedral (20-sided) head with a tail, 2) an icosahedral head without a tail, 3) and a filamentous form (Eiserling, 2023). Even though highly simplistic in structure, bacteriophages evolve to target a specific bacterium without impacting others. Such targeting specificity is ideal for delivery into the respiratory tract, where a large variety of bacteria reside (Stewart and Costerton, 2001). Another classification type mostly focuses on the bacteriophages’ tail components and is based on their biological cycle (Principi et al., 2019). According to this classification, there are two main groups: lytic or virulent, and lysogenic or temperate (Principi et al., 2019). Once either type enters the bacterial cell, the phage produces a viral genome and proteins instead of bacterial materials. Subsequently, the assembly and packing of the bacteriophages start, and the bacterial cells are lysed with the production of new virions that are ready to attack and infect other bacterial cells (Malik et al., 2017; Zhang et al., 2022). However, the process of cell bursting is implicated in the case of lytic bacteriophages. This process can vary according to the bacteriophage’s type, the pathogens against which the bacteriophage is focused, and the environments in which the bacteriophage–pathogen relationship arises (Chan et al., 2013).

Out of the estimated 1,030 phage particles, only a small number have been discovered and utilized to fight common bacterial infections in the lung.

#### 2.1.3 Streptococcus pneumoniae

Two types of bacteriophages, Dp-1 and Cpl-1, have been proven to reduce the number of *S. pneumoniae* bacteria along with other types of Streptococci (Mariano et al., 2016). Phage **Dp-1** was discovered earlier (Loeffler et al., 2001; López et al., 2004), but Cpl-1 seems to be more effective (Loeffler et al., 2003). For example, Cpl-1 is also delivered nasally, the nasopharynx being the area most infected by *S. pneumoniae* (Doehn et al., 2013). Severely ill female mice (C57Bl/6) received 25 mL of aerosolized Cpl-1; after 48 h, the endolysin decreased pulmonary bacterial counts and prevented bacteremia. Although concentrations of inflammatory cytokines were augmented just after Cpl-1 inhalation, the mice improved speedily, as shown by their surging body weight and inflammatory infiltrates fixed in the lungs, leading to a mortality reduction of 80% (Doehn et al., 2013). Moreover, the dosage of 15 mg/kg, of either Cpl-1 or Pal, does not show any increase in pro-inflammatory cytokine levels or significant changes in the fecal microbiome (Harhala et al., 2018). However, another bacteriophage appears to be more effective than Cpl-1. By intravenously injecting about 5 log10 CFU/mL of Lyta and Col-1, the former seems to kill about 80% of β-lactam-resistant (penicillin MIC, 2 μg/mL) meningeal pneumococcal isolate (strain MJD3693) (Rodríguez-Cerrato et al., 2007). Moreover, at 5 h post-challenge, the bacterial titers in the peritoneal fluid and the blood of the control animals were 7.4 ± 0.6 and 6.5 ± 0.7 log10 CFU/mL, respectively. Research on creating or discovering bacteriophages for *S. pneumonia* is ongoing, and, in recent years, a few new samples have been created. Examples are oral microbiome, 23 TH and SA01 (van der Kamp et al., 2020) and choline-binding proteins (CBPs) (Maestro and Sanz, 2016) and polypeptides found in pneumococcus, and include cell wall hydrolases, adhesins, and other virulence factors, which still need both *in vitro* and *in vivo* testing . For example, endolysin MSlys, a type of CBP, is shown within 2-h and 4mM to decrease planktonic cultures by 3.5 log10 CFU/mL, and 24-and 48-h-old biofilms by 1.5 and 1.8 log10 CFU/mL, respectively (Silva et al., 2020).

Another detailed study relates to ClyJ**,** a novel chimeric lysin. ClyJ is a putative lysin (gp20) that was encoded by the *Streptococcus* phage SPSL1 utilizing the LytA autolysin as a model. Molecular dissection of gp20 exposed a binding domain (GPB) comprising choline-binding repeats (CBRs) that are explicit for *S. pneumoniae*. ClyJwas then created by fusing GPB to the CHAP (cysteine, histidine-dependent amidohydrolase/peptidase) catalytic domain of the PlyC lysin. *In vivo* tests showed that there was no resistance in *S. pneumoniae* strains after exposure to incrementally doubling concentrations of ClyJ for 8 days (Yang et al., 2019).

#### 2.1.4 Haemophilus influenzae

Six types of bacteriophages have been discovered for this bacterium, such as HP1, S2A, B, C, N3, and φflu. Of these, only HP1 and three types of S2 have been investigated. HP1 is described as moderate and is thus unable to kill *H. influenzae* bacteria (Adamczyk-Popławska et al., 2020). Recently, another bacteriophage has been discovered—the N3 phage is only in NTHI strains and has a pattern distinct from HP1. However, poor detailed information is available on the bacteriophages for *H. influenzae* (Samuels and Clarke, 1969; Williams et al., 2002).

#### 2.1.5 Staphylococcus aureus

This is one of the strongest bacteria against the effect of several antibiotics. However, the search for a strong bacteriophage is still ongoing and very recent in the literature (Göller et al., 2021). The known bacteriophages of *S. aureus* are Caudovirales (tailed phages). If the tail is very short, the bacteriophages are called Podoviridae; if the tail is long and non-contractile, Siphoviridae; if the tail is long, contractile, and double-sheathed, Myoviridae (Xia and Wolz, 2014). Despite efforts in discovering and characterizing bacteriophages, most are inactive against *S. aureus*. A recent study developed JD219 that shows a broad host range able to contaminate 61 of 138 clinical strains of *S. aureus* tested, including the more virulent MRSA strains. The phage JD419 shows a peculiar morphology, with an elongated capsid and a flexible tail. The activity was maintained at pH values of 6.0–8.0 and below 50°C (Feng et al., 2021). Another bacteriophage pSa-3 shows some activity. Furthermore, its production can be boosted by increasing the bacterial inoculum and dropping the seeding phage MOI; this combination strategy could cut the phage production time (Kim et al., 2021). Alternatively, when inoculated into mice with *S. aureus* A170 (108 CFU/mouse), phage (109 PFU) rescued 97% of the mice; when applied to nonlethal (5 × 10^6^ CFU/mouse) 10-day infections, the phage (**M** ^**Sa**^) cleared the bacteria (Capparelli et al., 2007). The indication is that MSa can lyse only 7 of the 19 *S. aureus*. Moreover, this bacteriophage can act on bacteria formed for over 10 days (Capparelli et al., 2007). A newly discovered bacteriophage seems to kill *S. aureus*; however, it does not replicate, which is a major drawback for its replacement of antibiotics (Berryhill et al., 2021).

#### 2.1.6 Mycobacterium tuberculosis

This bacterium is highly worrisome due to its apparently strong resistance to the principal antibiotics. However, there are problems in the manufacturing of its phages (Palomino et al., 2008). Therefore, there is very little research on identifying and developing a bacteriophage that would act as an antibiotic. Even the limited references do not determine a phage but rather verify the resistance of *M. tuberculosis* to antibiotics (Froman et al., 1954). The few references focus on determining a bacteriophage usable for detecting *M. tuberculosis* in dairy or skin (Marei et al., 2003; Kalantri et al., 2005; Pai et al., 2005).

#### 2.1.7 Corynebacterium

The effect of bacteriophages on the virulence of this bacterium was proven several decades ago (Parsons and Frobisher, 1951); examples are β^tox+^, γ^tox−^, and L^tox+^ (Holmes and Barksdale, 1969). However, due to the small number of bacteriophages that were isolated and low interest in bacteriophages, research ceased (Carne, 1968).

#### 2.1.8 Neisseria

Several **Ngoϕfil** bacteriophages have been discovered to be active on *Neisseria* bacteria. There are four genetic islands on the surface of these bacteria, encrypting four filamentous phages: Ngoϕ6, Ngoϕ7, Ngoϕ8, and Ngoϕ9 (Kłyż and Piekarowicz, 2018). Only the first two have been shown to be aggressive to *Neisseria*. Filamentous Phage Ngoϕ6 can infect the cell with a mechanism regardless of the phage receptor and could infect different *Neisseria* bacteria. This page hosts PivNM/Irg recombinase, which is the entry for *Neisseria*. However, further studies are needed to test the *in vivo* and *in vitro* success of this phage.

#### 2.1.9 Fusobacterium

An icosahedral head and a segmented tail bacteriophage (Fnpϕ02) specifically infect this bacterium. This phage demonstrates a burst size of 100 phages per infected cell, determined along the 10-h rise period at 37°C (Machuca et al., 2010). FNU1 is another bacteriophage that acts against *Fusobacterium*. According to Kabwe et al. (2019), the median [Inter-Quartile Range (IQR)] absorbance at OD_600_ spectrophotometer of the biofilm of *Fusobacterium* was 2.17 (1.81–2.21), and with 24 h FNU1 treatment was 0.76 (0.71–0.89). Even for this bacterium, much work is still needed, and several investigations are required to determine a suitable bacteriophage.

#### 2.1.10 Moraxella catarrhalis

About 32 prophages have been derived from this bacterium; however, none have demonstrated an aggressive effect on it (de Vries et al., 2009; Ariff et al., 2015).

#### 2.1.11 Pseudomonas aeruginosa

Due to the aggressiveness of this bacterium, there is so much research that a review article summarizing them has recently been published (Chegini et al., 2020). Here, we report a brief list of all-mentioned bacteriophages acting against *P. aeruginosa*. One of the earliest discoveries was bacteriophage M-1, isolated from wastewater, that could eliminate biofilm caused by MDR isolates of *P. aeruginosa* in less than 6 h (Adnan et al., 2020). PB1-like, phiKZ-like, and LUZ24-like phages work against MDR *P. aeruginosa* under variable growth conditions, even against biofilms of *P. aeruginosa* (Latz et al., 2017). Pa193, Pa204, Pa222, and Pa223 eliminate the biofilm of *P. aeruginosa* isolated from patients with chronic rhinosinusitis with a single dose decrease the biofilm reproduction in 24 h (Fong et al., 2017). In addition, phage AZ1 can work against *P. aeruginosa* in planktonic and biofilm cells (Jamal et al., 2017). LysPA26, another bacteriophage, shows a higher antimicrobial activity at temperatures lower than 100°C (Guo et al., 2017). MAG1 and MAG4 can destroy 50% of the exposed bacteria—MAG4 has shown more lasting effects (Kwiatek et al., 2012). ФKMV, ФPA2, ФPaer4, and ФE2005 phages can also be effective against this bacteria, but *in vitro* and after 24 h (Mapes et al., 2016). The T7-like lytic phage (BVPaP-3) could constrain the biofilm formation (three logs) of hospital isolates of *P. aeruginosa* (Ahiwale et al., 2017). Lastly, phage PA1Ø is shown to be active against several Gram-negative and -positive bacteria, including *P. aeruginosa* (Kim et al., 2012).

Biofilms have often been expected to confer resistance on bacteriophages due to the impermeability of the biofilm matrix. However, even though they are far bigger than chemical antibiotics, bacteriophages are far smaller than their bacterial hosts, and many bacteriophages infect bacteria within biofilms. Therefore, there is potential for using bacteriophages against biofilm, but more investigations are necessary to materialize this potential (Stewart and Costerton, 2001; Harper et al., 2014).

### 2.2 Phage extraction and formulation

Bacteriophages can be stored in two different forms: liquid suspension or solid/dry powder formulation (Chang et al., 2018a). Either option has advantages and disadvantages and can impact the selection of the route of administration. Some considerations for selecting between them are target-specific delivery, phage stability, and clearance by the reticuloendothelial system of the recipient (Loc-Carrillo and Abedon, 2011). Developing optimal formulations for therapeutic phages is thus important for the efficacy and efficiency of phage therapy (Loc-Carrillo and Abedon, 2011). In fact, protein misfolding, denaturalization, and aggregation can affect bacteriophages, leading to a loss of functionality. These detrimental effects are commonly observed when the bacteriophages are exposed to adverse conditions, such as changes in pH, temperature, or moisture content (Parasion et al., 2014).

The conventional preparation of bacteriophages is as a liquid formulation, as it is for many other bioactive compounds. Liquid formulations are easy to prepare and have an average shelf half-life of about a year, depending on the type of phage. However, this method lacks accuracy; moreover, bacterial contaminants, such as exotoxins, endotoxins, or lipopolysaccharide (LPS) from the lysed cells, could damage the purity of bacteriophage formulations (Luong et al., 2020; João et al., 2021).

Despite the fact that the method for extracting bacteriophage in liquid form has been improved, refining this liquid extraction increases the costs and times (Hashemi et al., 2013; Hietala et al., 2013; Bonilla et al., 2016).

Regardless of the procedure followed to create the liquid formulation, a dramatic reduction in bacteriophage titer can occur at any time, depending on the individual stability of the phage (Malik et al., 2017).

**FIGURE 3:**
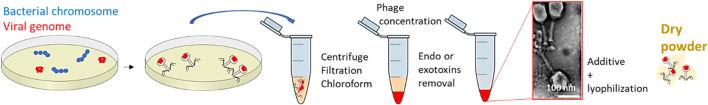
Procedure for preparing a solution of bacteriophages. Bacteriophage image represents the negative stain transmission electron micrograph of T4 phage processed through the PoT protocol (Bonilla et al., 2016), Copyright ^©^ 2016.

Dry powders, through lyophilization or other similar dehydration techniques, show a longer preservation of stability of bioactivity (Baldelli et al., 2022a). Such procedure is introduced in Figure 3. However, some dehydration processes need some additives, such as sugars, amino acids, proteins, and other components, to prevent osmotic damage or phage aggregation. Spray drying could also be used to prepare bacteriophage powders to be sold on the market. Details of spray drying will be shown in the following sections when discussing spray drying in encapsulation.

## 3 Encapsulation of bacteriophages

### 3.1 Particle engineering

Several drying techniques would allow the formation of dry powders. For example, thin film freeze drying (TFFD) (AboulFotouh et al., 2022) and lyophilization (Kawasaki et al., 2019) are some of the most straightforward techniques for generating dry powders for respiratory delivery. These, however, show some significant disadvantages for the purpose of proper encapsulation. First, a chemical procedure would be required to encapsulate bioactive compounds. This procedure can produce chemical waste, and be lengthy and costly (Peng et al., 2019). The main drawback of TFFD is its extremely long processing time, which would not allow it to be transferred into large-scale production (Zhang et al., 2020; Ergin, 2022). A bacteriophage has been successfully encapsulated via lyophilization: Golshahi et al. (2011) encapsulated the phages S4-M and ϕKZ for *Burkholderia cepacia* and *Pseudomonas aeruginosa* using a mixture of lactose and lactoferrin at 60: 40 w⁄w. However, powders produced by lyophilization tend to have a broad size distribution, resulting in poor target delivery (Shekunov et al., 2007). About 15%–30% of the particles show an average size of 5–0.5 µm; this results in only 45% of the powder reaching areas beyond the throat in the respiratory tract (Golshahi et al., 2011).

**FIGURE 4:**
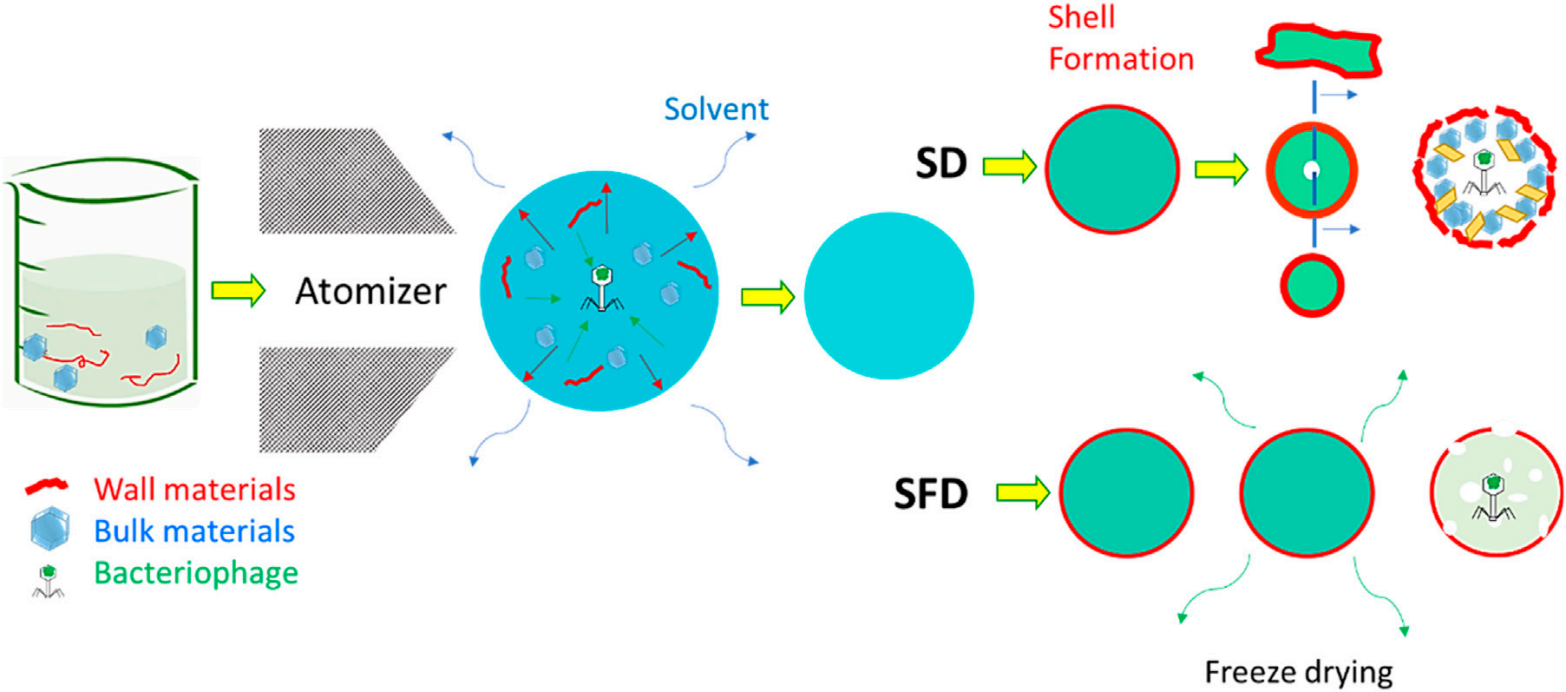
Comparison between spray and spray freeze drying for the encapsulation of bacteriophages. The presence of both wall and bulk materials supports this encapsulation. The images have been adjusted from a previous publication (Baldelli et al., 2022a). Copyrights obtained from ^©^ 2022 Elsevier Ltd.

Particle engineering theory can assist spray drying and the prevention of damage (Vehring et al., 2007; Vehring, 2008; Baldelli et al., 2022c; Baldelli et al., 2023). The theory of particle engineering explains the formation process of solid micro- and nanoparticles from liquid droplets (Baldelli and Vehring, 2016b). An extensive review article describes the parameters influencing particle formation; for example, temperature, airflow, atomizer diameter, and percentages of the chemical compounds can all influence dry powders’ chemical and morphological properties (Shoyele and Cawthorne, 2006; Vehring et al., 2007; Baldelli et al., 2016; Ferraz-Albani et al., 2017; Baldelli et al., 2022b). Particle engineering is thus the leading theory of some of the most common drying technologies, such as spray drying (SD) (Vehring et al., 2007) and spray freeze drying (SFD) (Baldelli et al., 2022d), as shown in Figure 4. The main difference between the former and latter is the operating temperature: SFD allows colder temperatures (Baldelli et al., 2022d). These drying techniques have been used for several decades to produce dry powders to deliver bioactive compounds to the respiratory system (Baldelli et al., 2022a). SD is the most common technique used for producing dry powders containing encapsulated bioactive compounds for respiratory delivery. Due to the average size of SD microparticles, the lung is the most common deposition location of SD powders. The smaller the diameter of SD microparticles, the deeper in the respiratory system they can be deposited (Baldelli et al., 2015).

Spray drying procedures tend to apply thermal energy to the evaporating solvent for a few milliseconds. This thermal stress is commonly intense, though limited, but can last throughout the spraying procedure; even at lower temperatures than the inlet ones, a process taking a few seconds can jeopardize the stability of bioactive compounds. Another possible source of damage can be shear stress, which occurs when spraying the liquid feed over the nozzle and atomizing it into small droplets (Ma et al., 2015). To bypass these types of stress, and thus the possibility of damaging the bacteriophage during the delivery process, encapsulation can be used using SD or SFD. This can be achieved by adding additional materials to spray the formulation. These materials have been called “wall” and “bulk” (Baldelli et al., 2022d; Ahad et al., 2023; Baldelli et al., 2023; Nimbkar et al., 2023). Wall material is the outer layer of micro- or nanoparticles containing an encapsulated compound. The purpose of this material is obvious: it creates a shell that covers and protects a bioactive compound. This shell is useful for reasons other than the increase of bioavailability of the encapsulated bioactive compound. The wall material is usually a polymer with a higher molecular weight than the bioactive compound to be encapsulated. This characteristic allows the formation of the shell at an early stage in the particle formation process (Baldelli et al., 2015). Forming a shell for each sprayed droplet ensures a high bioavailability of the bioactive compound and, thus, a long half-shell life (Risch, 1995; Baldelli et al., 2022a). The properties of the dry powder are highly dependent on the properties of the shell (Shamaei et al., 2017). For instance, if the polymer is known to show mucoadhesive properties, then the dry powders are expected to have a high residence time on the mucosa in the respiratory tract (Baldelli et al., 2022b). Adhesion forces can also be influenced by the type of material used to form the spray-dried microparticle wall (Fahs et al., 2010). However, the roughness of the microparticles can impact the adhesion forces. This property can be tuned by both the wall material, the spraying conditions, and the presence of a bulk material (Baldelli and Vehring, 2016b). The bulk material is considered a cushion for the bioactive compound for protection greater than that provided by a shell alone. Depending on the quantity, the presence of a bulk material has a great influence on the morphology of spray-dried microparticles (Baldelli et al., 2022a). In some cases, wall and bulk materials are not the only components of a spray-dried formation to encapsulate bioactive compounds—amino acids, such as leucine, are widely used to encapsulate a bioactive compound via spraying techniques (Lechuga-Ballesteros et al., 2008; Ordoubadi et al., 2021; Wang et al., 2021; Baldelli et al., 2022a). The use of amino acids is justified by the promotion of shell formation. Previous research has found that the presence of leucine, at weight percentages between 6 and 22, facilitates shell formation in a variety of spray-dried formulations (Chow et al., 2017). Leucine recrystallizes on the surface of the particles, generating a shell to decrease inter-particle interaction and boost the dispersibility of the powder (Leung et al., 2016). Other amino acids have been used, but there is poor available knowledge on the comparison of these other amino acids with leucine (Carrigy et al., 2020). This review article on designing encapsulated bacteriophage thus focuses on the selection of bulk and wall materials.

### 3.2 Selection of the impacting parameters

While the use of bacteriophages in treating bacterial infections is well-established, the use of spraying techniques as a method for encapsulating bacteriophages for nose or lung delivery is fairly recent. Therefore, the encapsulation of bacteriophages using spraying techniques has been demonstrated in only a limited quantity of research (Table 1). Most of the references cited in Table 1 use a common laboratory-scale spray dryer, Buchi 290, with an atomizer nozzle of 0.6–0.7 mm in diameter. In addition to Buchi 290, other similar products, such as Buchi 191 and 90, were used in previous studies. The reason for this common use is the popularity of this spray dryer and its ability to scale up results (Vehring et al., 2007). In addition, spray drying produces microparticles of about 0.5–20 μm, which is suitable for delivery to the respiratory tract (Baldelli et al., 2015). Furthermore, twin-fluid atomizers have been shown to create lower stress than other types, such as vibrational ones, on the encapsulation of bacteriophages (Carrigy et al., 2020). Another connection between most previous research on the encapsulation of bacteriophages for respiratory delivery is the target: about 90% of previous research focuses on *P. aeruginosa*. The phages of this bacterium seem to be more stable than others; for instance, they appear stable for temperatures between 25°C and 50°C and pH between 3 and 11 (Akremi et al., 2022). In addition, the antibiotic resistance of *P. aeruginosa* is one of the highest, attracting the attention of the research community (Golshahi et al., 2011).

**TABLE 1:** List of research that creates encapsulated dry powder for delivery to the respiratory system of bacteriophages. Type of phage, target, method, and materials used are shown. The number of phages used in the following references varies between 10^9^ and 10^10^ PFU/m. FR an

Phage	Target	Method		Material	Result	Reference
Bacteriophage	Bacterium	Temp	Flow rate	Bulk		
Myoviridae (KS4- M, KS14, ϕKZ/D3 and ϕKZ/D3/KS4-)	*Burkholderia cepacia* and *P. aeruginosa*	Inlet = 75°C and Outlet = 42°C	Feed, 0.33 mL/min and gas, 100 L/min	Trehalose, leucine, and casein sodium salt, and surfactants as tyloxapol or pluronic	All formulations show lung deposition of at least 10^6^ pfu	Matinkhoo et al. (2011)
*Pseudomonas* phage LUZ19 and *Staphylococcus* phage Romulus	*P. aeruginosa PAO1* and *S. aureus*	Inlet = 85°C and 100°C	Feed = 2 mL/min, and an gas = 6 L/min	Lactose, trehalose, or dextran 35	Trehalose-containing phage particles show only 1 logarithmic unit reduction in phage titer	Vandenheuvel et al. (2013)
Bacteriophage virus-like particle (VLP)	NA	Inlet = 145°C, outlet = 50°C	Feed = 3 mL/min, gas = 11 L/min and liquid	D-mannitol and L-leucine	Dry powder VLP is stable for 1 year at 37°C	Saboo et al. (2016)
Myoviruses and podoviruses (PEV1, PEV20 and PEV61)	*P. aeruginosa*	Inlet = 60°C, outlet = 40°C	Feed 1.8 mL/min and a gas = 12 L/min	Trehalose, lactose, mannitol, glycine, leucine, and Pluronic F68	Phages spray dried with 12 mg/mL trehalose or lactose resulted in less than log_10_ 1.5 titer loss	Chang et al. (2017)
N4-type, lytic podovirus, PEV2	*P. aeruginosa*	Inlet = 60°C, outlet = 40°C–45°C	Feed 0.02 mL/min and gas of 13 L/min	Trehalose, mannitol, and leucine	Trehalose with content higher than 40% shows less than 1.3 titer reduction	Leung et al. (2017)
*Pseudomonas* phage PEV20 10^10^ PFU/m	*P. aeruginosa*	Inlet = 60°C, outlet = 40°C	Feed 1.8 mL/min and gas of 13 L/min	17 mg/mL of lactose and 8 mg/mL of leucine	PEV20 with lactose and leucine-produced powder with 2 × 10^7^ PFU/mg	Chang et al. (2018b)
*Pseudomonas* phages PEV2 PEV1 and PEV20	*P. aeruginosa*	Inlet = 60°C, outlet = 40°C	Feed 1.8 mL/min- gas of 13 L/min	Lactose 80% (wt/wt) and leucine 20% (wt/wt)	Mild titer reduction (ranging 0.11–1.3 logs)	Li et al. (2021)
*Pseudomonas* phage PEV20 10^10^ PFU/m	*P. aeruginosa*	Inlet = 60°C, outlet = 40°C	Feed 1.8 mL/min and gas of 13 L/min	Lactose (8–19 mg/mL) and leucine 8 mg/mL)	PEV20 powder with lactose and leucine stable for 1 year at 20°C and 15% RH	Lin et al. (2019)
Bacteriophage L2 virus-like particle (VLP)	NA^a^	NA	NA	85.4% mannitol, 1.71% trehalose, 0.85% dextran, 7.85% L-leucine, and 4.27% inositol	Thermostability and protective efficiency of spray-dried MS2-16L2 VLPs after storage for 34 months	Peabody et al. (2017)
Phage D29	*M. tuberculosis*	Inlet = −130°C, outlet = −80°C	Feed 20 mL/min and gas of 10 L/min	Trehalose and mannitol in varying concentrations	7:3 trehalose and mannitol for cryoprotection of phages	Ly et al. (2019) ^.^ ^b^
Podovirus PAO1K	*P. aeruginosa*	NA	Feed 2 mL/min and gas of 10 L/min	Trehalose and isoleucine 60:40, 70:30, 80:20, and 90:10% w/w	Increase in isoleucine content decreases bioavailability	Tabare et al. (2021)
Lytic Myoviridae phage, AB406	*A. baumannii*	Inlet = 60°C, outlet = 40°C	Feed 1.8 mL/min and gas of 13 L/min	Trehalose to mannitol ratio (80:0, 60:20, and 40:40) and leucine 20%	Powders with mannitol >60 mg/mL show no titer loss for a month	Yan et al. (2021) ^.^ ^c^

^a^ Aimed for papillomaviruses (HPVs).

^b^ Use of atmospheric spray freeze drying.

^c^ Instead of Buchi 290, it used Pilotech YC-500, spray dryer.

Regardless of the target, the number of bacteriophages used needs to be above 10^10^ pfu/mL since, assuming possible losses during the encapsulation procedure, the required quantity for an effective treatment against most respirator bacteria is 10^6^ pfu/mL (Su et al., 1998; Danelishvili et al., 2006; Chhibber et al., 2008; Chegini et al., 2020). The optimization of the encapsulation procedure could potentially reduce the losses of the bacteriophages. Such optimization can be achieved by tuning the atomization conditions and the materials selected. Having the greatest effect on the stability of sprayed bioactive compounds are the atomization liquid and airflow speed rates and the spraying temperatures (Baldelli et al., 2022e). The gas flow levels of the feed are between 1.8 and 3 mL/min and of the gas are between 11 and 13 L/min. Within these values, bacteriophages appear not to undergo the lowest stress. Lower levels of feed flow rate do not allow the liquid to form droplets and evaporate into dry microparticles (Sheu and Rosenberg, 1995). Higher levels of gas flow rate can reduce the spraying yields and increase the stress applied to bacteriophages (Carrasquillo et al., 2001). An elevated atomizing airflow (12 compared 5 L/min) showed a higher reduction of phage titer (Vandenheuvel et al., 2013).

The lower the temperature, the less damage occurs to a bioactive compound through a spraying procedure (Baldelli et al., 2022d). Bacteriophages have been encapsulated using spray drying at temperatures of the following ranges: inlet at 60°C and outlet at 40°C. For example, one of the lowest titer reductions (0.11) of *P. aeruginosa* phages has been achieved using these inlet and outlet temperature conditions (Li et al., 2021).

Atomization conditions can have an impact on the stability of bioactive compounds, but the efficiency of the encapsulation procedure is mostly dependent on material selection. When no materials are added to the formulation of bacteriophages, the lowest titer reduction is achieved. Therefore, adding another component to the matrix ensures the survival of most of the bacteriophages. Sugars have been the most common additive used (Shoyele and Cawthorne, 2006). Without these, spray-dried phages were unable to retain their bioactivity during the spray drying process, resulting in log10 6.7 loss or complete titer reduction [118]. In addition, sugars and carbohydrates are able to replace the bioactive compound water hydrogen bonds due to their relatively high molecular weight and structure (Chang et al., 2017).

The most popular sugars are trehalose, mannitol, and lactose, which are also the most commonly used in respiratory delivery (Vehring, 2008). For instance, lactose and trehalose are commonly used to shield biomaterials against desiccation in spray drying. Both saccharides show a high glass transition temperature when in anhydrous amorphous form (108°C and 115°C for lactose and trehalose, respectively). For instance, the majority of PEV1, PEV20, and PEV61 phages spray dried with 12 mg/mL trehalose or lactose resulted in less than log 10 1.5 titer loss (Chang et al., 2017). However, amorphous lactose is hygroscopic; *T*_g_ decreases when exposed to succeeding uptake of moisture. The hygroscopic nature of amorphous lactose could generate a lack of phage protection. Amorphous powders are thermodynamically unstable; they can, thus, recrystallize when exposed to moisture (Chang et al., 2017). Thus, trehalose seems more efficient as a stabilizer since it is not influenced by high residual moisture contents (Vandenheuvel et al., 2013). When trehalose is used as an excipient when drying a bioactive compound, the compound's immobilization in a glassy matrix hinders the denaturalization of the bioactive compound. Furthermore, trehalose is highly attracted to water and can act as a lyoprotectant (Ly et al., 2019). For instance, after spray drying, *P. aeruginosa’s* phage shows a total 1.3 log titer reduction in formulations containing 40%, 60%, and 80% trehalose, and 2.4 and 5.1 log reductions in formulations containing 20% and no trehalose, respectively (Leung et al., 2017).

Although the use of lactose alone seems to be not as functional as trehalose in encapsulating bioactive compounds, the addition of leucine makes the lactose–leucine combination efficient. Leucine is a hydrophobic amino acid that is often used in spray-dried and respirable formulations to enhance powder flowability and dispersibility (Wang et al., 2022). Moreover, leucine can also influence stability against moisture for spray-dried inhalable powders stored at elevated humidities. In the evaporation process, the high hydrophobicity of leucine allows its distribution on the surface, forming a crystalline shell and enhancing the protection of the bacteriophage. For example, spray-dried PEV20 powder containing lactose and L-leucine remains stable for 1 year at 20°C when stored inside an aluminum pouch (Lin et al., 2018). Furthermore, the highest lung dose is obtained with solutions involving 17 mg/mL of lactose and 8 mg/mL of leucine for PEV1 and PEV61, and 20 mg/mL of lactose and 5 mg/mL of leucine for PEV20 (mostly because of the morphology) (Chang et al., 2017). The combination of lactose, trehalose, and leucine can further enhance the protection of the bioactive compound. Formulations containing 40% trehalose, 40% mannitol, and 20% leucine had better storage stability, under storage conditions of −4°C and 30% relative humidity, of *P. aeruginosa* phage with no further phage loss after 1 month and <1 log storage loss (Yan et al., 2021). The presence of leucine also supports the storage and of phages. Spray-dried powder containing 80% lactose, 20% leucine, and podovirus phage can be stored and handled below 60% RH to prevent crystallization; they stay stable for 1 year at 15% humidity and humidity 20° (Yan et al., 2021).

Other materials used in previous research focusing on encapsulation via spray drying of bacteriophages are mannitol, dextran, and pullulan. Of these, dextran has the highest molecular weight, allowing its distribution across the surface of an evaporating droplet, thus forming a shell. Moreover, dextran is used for spray drying biological materials, preventing *P. aeruginosa* from connecting to lung epithelial cells and for improving CF sputum clearance (Chang et al., 2021).

### 3.3 Overall suggestions for bacteriophage encapsulation

Spray drying is the leading technique in encapsulating a bacteriophage for respiratory delivery. The relevant conditions are the inlet and outlet temperatures. For any bacteriophage, these should be below 80°C and 40°C, respectively. When using water as a solvent, it is a challenge to ensure the formation of a shell during the particle formation process. Therefore, as explained in the previous section, leucine, and similar amino acids are valid alternatives since they have been shown to promote shell formation. Together with an amino acid, a common selection is to use a sugar, such as lactose, sucrose, trehalose, or mannitol. Lactose and sucrose have low glass transition temperatures and tend to be more susceptible to crystallization upon room temperature storage (Thiyagarajan et al., 2021). Avoiding crystallization could be beneficial to reducing the risk of damaging the phages. Therefore, as demonstrated by previous research (Ma et al., 2015), trehalose could be a valid option. However, mannitol could be another option when both the procedure and storage temperatures are kept above water’s freezing point. At warm temperatures, mannitol tends not to crystallize (Littringer et al., 2012). The combination of mannitol and trehalose appears to be favorable for phage encapsulation. As a crystalline bulking agent, mannitol can avert the failure of a powder cake, which facilitates drying at higher temperatures and acts as an excipient in inhibiting trehalose from crystallizing (Su et al., 2017). Mannitol is projected to crystallize during the drying stage and form a supporting skeleton for the particles to avoid further mobility of the trehalose particle structure when drying (Chang et al., 2021).

The amount of sugar needed may vary according to the shape and length of the phages. The larger the phage, the more material might be needed to preserve the phage’s bioavailability. For instance, lytic bacteriophages Dp-1 and Cp-1 are roughly 6 µm long. Spray-dried microparticles larger than this would be expected in order to enclose the bacteriophage without major damage. However, a wall material could be used to enhance the protection of the bacteriophages. This could complicate the formulation matrix, but it could ensure shell formation, control the adhesion forces between multiple sprayed microparticles, and improve adhesion to the mucus layer in the respiratory tract (Baldelli and Vehring, 2016b). Moreover, the presence of a hydrophobic polymer on the surface of sprayed microparticles could reduce the impact of humidity on the half-shelf life of powders. In particular, a thicker layer on the surface could increase half-shelf life (Vandenheuvel et al., 2013; Baldelli et al., 2023). Humidity causes the crystallization of any amorphous matrix, ruining the embedded phages. Therefore, reducing its effect on the phages could greatly enhance the half-shelf life of powders. Another process absent in the literature is spray freeze drying. Even though this can be more challenging to design and scale up than spray drying, it can maintain the procedure’s temperatures below 10°C. These low temperatures commonly show a lower chance of jeopardizing the bioavailability of bioactive compounds (Baldelli et al., 2022d).

## 4 Conclusion

Resistance to antibiotics affecting the respiratory tract has drastically increased. For instance, *Streptococcus pneumoniae* shows almost 100% strain resistance to the popular antibiotics penicillin and cefactor. Bacteriophages can bypass such resistance and kill bacteria and biofilms in the respiratory tract. In the case of *Staphylococcus aureus*, the most common bacteriophage type is Caudovirales. For instance, a type of Caudovirales (phage (109 PFU) has been shown to fully eradicate the infection in mice (5 × 10^6^ CFU/mouse). Although bacteriophages are high efficient at targeting and killing specific types of bacteria, they show low efficacy when delivered to the respiratory system. The encapsulation of bacteriophages can support improved deposition location in the respiratory tract and, thus, the drug’s efficacy. Spray drying is the leading technique for creating encapsulated bioactive compounds. However, due to the high sensitivity of bacteriophages, spray freeze drying might be more appropriate due to the lower stress applied to the bioactive compound. However, the size of the particle formed by spray freeze drying can be applied mostly to nasal delivery. For most types of bacteriophage, leucine or an alternative amino acid is suggested for promoting particle formation. Furthermore, a bulk material is recommended for further protection of the encapsulated compound and for the design of the microparticles. A combination of trehalose and mannitol seems the optimal solution since it avoids any crystallization effect which might jeopardize the stability of the encapsulated bacteriophage. Due to the limited research literature available on the encapsulation of bacteriophages for delivery into the respiratory tract, these conclusions can be considered solely as assumptions.
